# A harmonic current detection algorithm for aviation active power filter based on generalized delayed signal superposition

**DOI:** 10.1038/s41598-025-94829-x

**Published:** 2025-03-26

**Authors:** Yong Lu, Bohan Li, Guofei Teng, Zhen Zhang, Xianfeng Xu

**Affiliations:** 1 School of Energy and Electrical Engineering, Chang’an University, Xi’an, 710064 China; 2 Power Technology Laboratory, AVIC Computing Technique Research Institute, Xi’an, 710000 China

**Keywords:** More electric aircraft, Variable-frequency-grids, Active power filter, Harmonic detection method, Generalized delayed signal superposition, Energy science and technology, Engineering

## Abstract

To address the limitations of traditional harmonic detection methods for active power filters in variable-frequency-grids of the More Electric Aircraft (MEA), including inadequate filtering performance and poor adaptability to frequency variations, this paper proposed a harmonic detection algorithm tailored for active power filters in variable-frequency-grids of the MEA. Firstly, the filtering principle of the proposed operator is derived in detail, and its filtering characteristics and frequency adaptability are analyzed. Secondly, based on the proposed operator, a comprehensive harmonic current detection algorithm is designed to accommodate the wide frequency variation of variable-frequency-grids. Then, the complexity of the operator was analyzed. Due to the symmetry of the coefficients, the operation time of the operators can be reduced. Finally, experimental validation and comparative analysis with traditional harmonic detection methods are conducted. Experimental results demonstrated that the proposed harmonic current detection algorithm effectively detects harmonics in complex distorted current signals of aerospace power systems, confirming the algorithm’s accuracy, superior performance, strong robustness and practical applicability.

## Introduction

To achieve higher fuel efficiency and lower carbon dioxide emissions, the concept of (MEA) has garnered significant attention in the aviation industry^1,2^. MEA technology replaces multiple secondary energy sources in traditional aircraft with electrical energy, thereby enhancing energy conversion efficiency, power density, and overall aircraft performance^3^. The power system serves as the energy source for MEA, and a stable and reliable power system is crucial to ensuring the safe operation of the aircraft^4^. Variable-frequency-grids of the MEA, which output AC power with frequencies ranging from 380 to 800 Hz, significantly reduce the weight of onboard equipment^5^. These systems feature a simple primary power structure, high energy conversion efficiency, lightweight design, high reliability, and ease of operation and maintenance, making them a key developmental direction for future MEA power systems^6^.

**Fig. 1:**
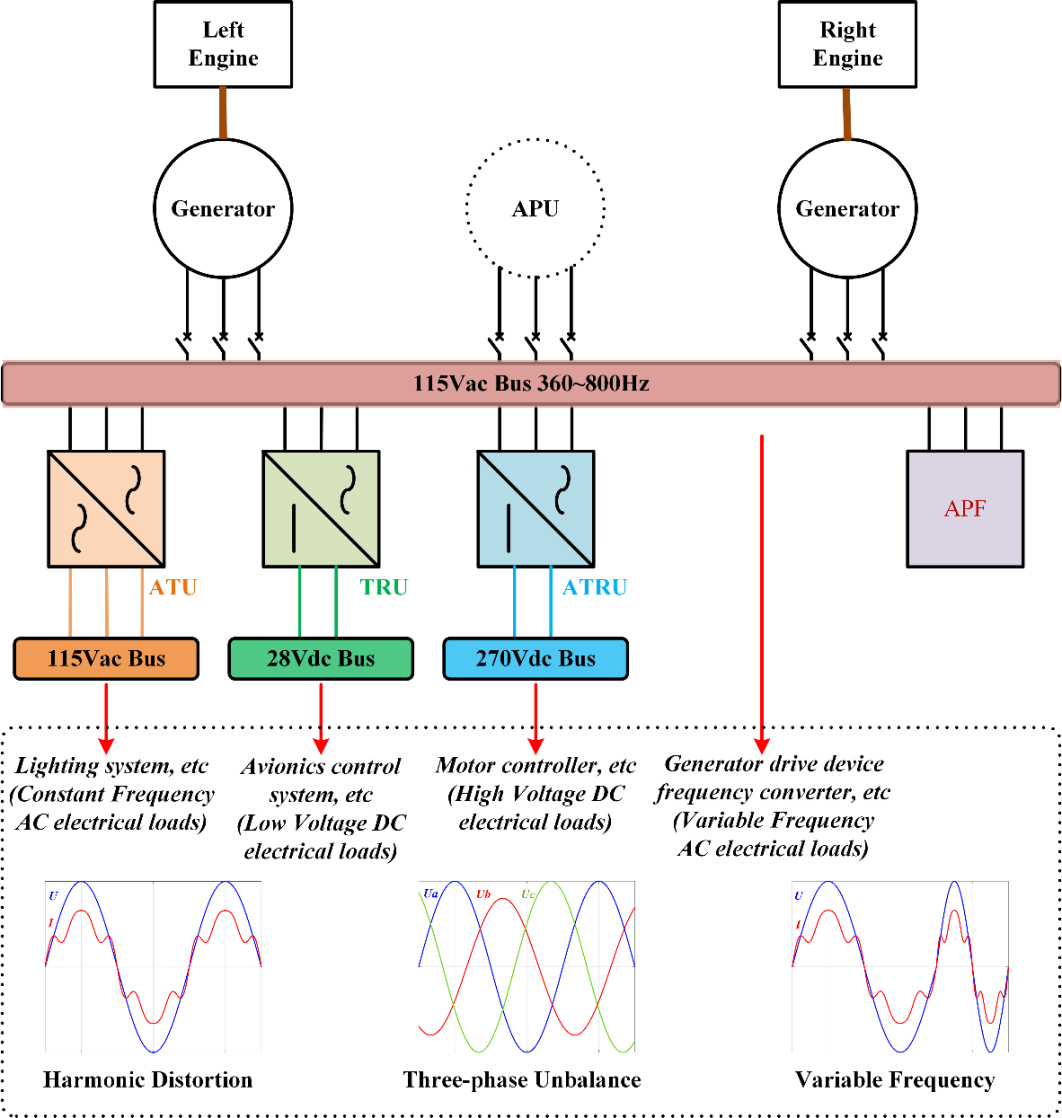
Schematic diagram of MEA’s variable-frequency grid power system.

As shown in Fig. 1, a typical variable-frequency-grid of MEA includes various energy conversion components, such as the autotransformer unit (ATU), transformer rectifier unit (TRU), autotransformer rectifier unit (ATRU). These components are key components of MEA energy conversion, but they are essentially nonlinear conversion devices based on power electronic devices. In addition, the widespread use of nonlinear aerospace electronic loads in variable-frequency grids presents significant challenges. These challenges include the generation of numerous harmonics, three-phase imbalance, and substantial frequency variations in grid currents, all of which degrade the power quality and reliability of the system^7,8^. Such issues can pose considerable risks to the flight safety and stable operation of the aircraft. Therefore, ensuring the safety and reliability of the MEA’s variable-frequency-grids, along with improving power quality, is of paramount importance^9^.

**Fig. 2:**
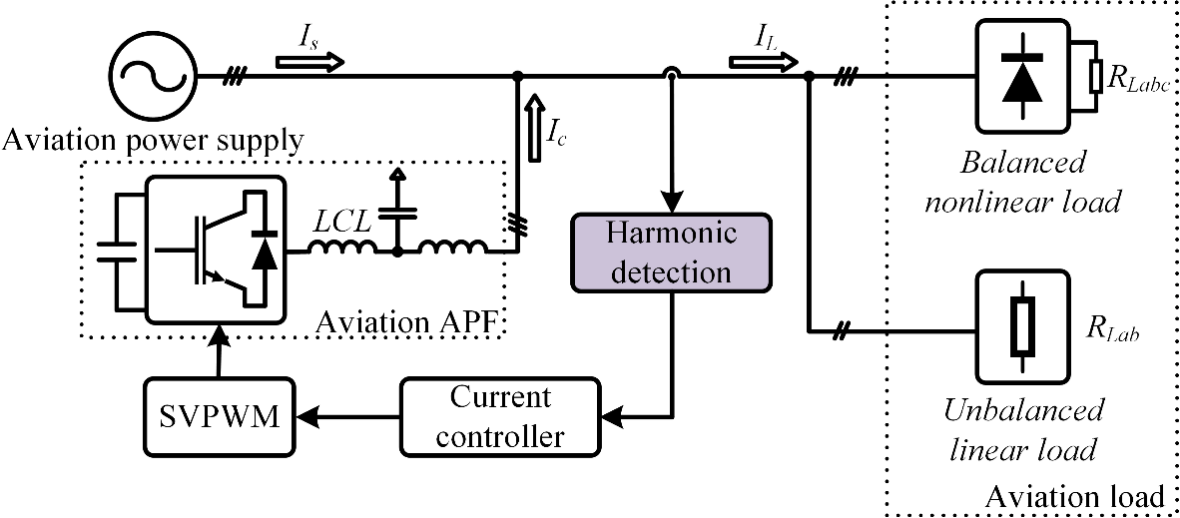
Schematic diagram of aviation active power filter.

Active Power Filters (APF) are essential onboard devices for MEA, designed to dynamically compensate harmonics and improve power quality, thereby addressing harmonic pollution in variable-frequency-grids of the MEA and enhancing the reliability of power supply^10^. A key component of APF operation is the harmonic current detection process, as the accuracy and speed of the detection algorithm directly determine the effectiveness of harmonic compensation^11^. Figure 2 illustrates the structure of aviation active power filter. Given that load currents fluctuate with varying load conditions, harmonic current detection algorithms typically isolate the fundamental component from the total load current. The harmonic current is then determined by subtracting the fundamental component from the overall load current, enabling accurate detection of harmonic distortions.

In variable-frequency-grids, common time domain harmonic current detection methods include: *i*_*p*_*-i*_*q*_ method based on instantaneous reactive power theory^12^. This method has a simple structure, which transforms the input signal into synchronous rotating frame through park transform, and uses a fixed cut-off frequency low-pass filter (LPF) to filter the harmonic signal, which has a certain frequency adaptability. P. Kanjiya et al. proposed an adaptive low pass filter (ALPF) with variable cut-off frequency to overcome the disadvantage of fixed cut-off frequency in LPF^13^, but this method has poor filtering ability and long dynamic time in complex power grid environment^14^. Harmonic detection method based on moving average filter (MAF)^15^, this method used MAF to replace the LPF in *i*_*p*_*-i*_*q*_ method, and improved the filtering performance, but has a fixed average window length. E. Robles et al. proposed a MAF filter with variable average window length to enhance the frequency adaptability^16^, but it has more coordinate transformation links and complex structure. G. Panda et al. used a phase-locked loop (PLL) to update the average window length, but did not consider dynamic response^17^. Harmonic current detection method based on adaptive notch filter (ANF)^18,19^. This method can detect each harmonic component in the input signal without PLL. However, the parameters in ANF structure are often fixed, which limits its application. Harmonic current detection method based on sinusoidal amplitude integrator (SAI)^20^, which realized frequency adaptation through PLL, has poor filtering performance when the harmonic content of power grid is high. Harmonic current detection method based on cascaded delayed signal cancellation (CDSC)^21^, which required many delayed signal cancellations (DSCs), has poor real-time performance and is not suitable for aviation APF. The harmonic detection method in frequency domain is usually based on the Fourier transform. E. Lavopa et al. proposed a real-time implementation of Discrete Fourier Transform (DFT)^22^, which can effectively detect the fundamental and harmonic components when the frequency changes. V. I. Suryawanshi et al. proposed a smart discrete Fourier transforms (SDFT) based algorithm to solve the calculation error problem caused by the traditional Fourier transform in the case of frequency change^23^. But these methods have a large amount of calculation. To reduce the computational complexity, B. Yang et al. proposed a recursive discrete Fourier transform (RDFT) based algorithm^24^. This method reduced the computational complexity, but the selectivity of RDFT algorithm also decreases. K. Selvajyoth et al. proposed composite observers to detect harmonics, but the dimension of the system is relatively large and has certain conservativeness^25^. Guojing Li et al. proposed a harmonic detection algorithm based on the optimize reduced order observer, this method optimizes the number of projects to be estimated. However, it requires prior knowledge of the frequency during design^26^. Table 1 presents the Total Harmonic Distortion (THD) results of the literature (References [12,14,15,20,24]) in harmonic condition.

**Table 1:** Comparative analysis of THD performance across mentioned methods.

Mentioned methods	THD results
*i*_*p*_*-i*_*q*_^12^	5.95%
MAF^14^	7.4%
dq-frame CDSC (dqCDSC)^14^	8.7%
ALPF Based dqCDSC^14^	6.4%
ALPF Based MAF^14^	5.8%
Adaptive Bandpass Filter (ABPF) based dqCDSC^14^	2.7%
ABPF based MAF^14^	0.5%
Heterodyning, MAF, and PLL^15^	3.50%
SAI^20^	3.04%
RDFT^24^	4.0%
Twice-sampling (TS)-RDFT^24^	2.2%

The variable-frequency-grids of MEA are complex and dynamic, posing significant challenges for traditional harmonic detection methods, which often exhibit poor accuracy and speed when applied to aerospace active power filters. The harmonic current detection method utilizing a LPF depends heavily on the selection of the LPF cutoff frequency. This cutoff frequency must strike a balance between achieving a fast dynamic response and maintaining detection accuracy. However, this trade-off presents a challenge in meeting the demands for rapid tracking of high frequency signals with frequency variations, particularly in MEA’s variable-frequency-grids where fast and accurate detection is essential. Other time domain harmonic detection methods, such as harmonic detection based on ANF and SAI, rely on precise and adaptive adjustment of the filter notch frequency and bandwidth. Their performance tends to degrade when subjected to large frequency deviations or unbalanced power grid conditions, making them less reliable in dynamic or irregular operating environments. Additionally, the improved harmonic current detection method based on DFT and mathematical matrix calculations generally suffer from the problem of large computational complexity and are not suitable for application in aviation active power filters.

To address these challenges, this paper proposes a novel harmonic current detection method based on Generalized Delayed Signal Superposition (GDSS) operators, specifically tailored to the characteristics of MEA’s variable-frequency grids. Firstly, two GDSS operators with strong frequency-selective properties and rapid dynamic response are derived using an algebraic recursive approach. These operators are capable of accurately and swiftly extracting the fundamental component from distorted input signals, generating a pair of clean, orthogonal signals. Secondly, the system frequency is continuously monitored through a PLL, enabling real-time adjustment to variations in input frequency. Simultaneously, fractional delay interpolation is achieved using Lagrange interpolation polynomials, significantly enhancing the adaptability and accuracy of the GDSS algorithm under varying frequency conditions. Thirdly, a positive-sequence separation structure is introduced to isolate the positive-sequence fundamental component in the context of a three-phase unbalanced power grid. Finally, Experimental validation and comparative analysis with traditional harmonic detection methods were carried out under four different conditions: harmonic distortion, three-phase imbalance, frequency jump, and complex distortion. The experimental results demonstrate that the proposed detection algorithm exhibits both high accuracy and fast dynamic response while maintaining strong robustness. It can rapidly and accurately extract harmonic currents in various complex environments, offering an effective solution for harmonic detection in dynamic and variable-frequency MEA power grids. This significantly enhances the practicality of Active Power Filters (APFs) in aviation power systems.

## The proposed GDSS operators

### Analysis principle algorithm

When current contains harmonics, the current signal can be decomposed into a combination of a fundamental component with a frequency of *ω* and harmonic components with frequencies of *hω*. The mathematical expression for this decomposition is as follows:

1 $$i(t) = \sum\limits_{h = 1}^{H} {I_{h} \cos (h\omega t + \varphi_{h} )}$$

where *h* is harmonic order(for fundamental component, *h* = 1), *H* represents the maximum harmonic order, *ω* and *φ*_*h*_ are the fundamental frequency and initial phase of the *h*-order harmonics, respectively. Two different types of generalized delayed signal operators (GDS1 and GDS2) for *i*(*t*) are considered in this paper:

2 $$GDS1[i(t)] = i(t - \frac{k}{{h_{s} n}}T)\cos \frac{2k\pi }{n}$$

3 $$GDS2[i(t)] = i(t - \frac{k}{{h_{s} n}}T)\sin \frac{2k\pi }{n}$$

where *T* is the fundamental period, *h*_*s*_ is the harmonic order to be detected, and *n*, *k* are arbitrary integers. If *GDS*1[*i*(*t*)] and *GDS*2[*i*(*t*)] are added with *k* ranging from 0 to *m*(*m* < *h*_*s*_*n*) and then multiplied by 2/(*m* + 1), two types of GDSS operators can be constructed as follows:

4 $$GDSS1[i(t)] = \frac{2}{m + 1}\sum\limits_{k = 0}^{m} {i(t - \frac{k}{{h_{s} n}}T)\cos (\frac{2k\pi }{n})}$$

5 $$GDSS2[i(t)] = \frac{2}{m + 1}\sum\limits_{k = 0}^{m} {i(t - \frac{k}{{h_{s} n}}T)\sin (\frac{2k\pi }{n})}$$

Considering the *h*-order harmonic signal $i_{h} (t) = I_{h} \cos (h\omega t + \varphi_{h} )$ in $i\left( t \right)$, Eq. (4) can be expressed as Eq. (6). By applying the product-to-sum trigonometric identity, Eq. (6) can be rearranged to be Eq. (7).

6 $$GDSS1[i_{h} (t)] = 2\frac{{I_{h} }}{m + 1}\sum\limits_{k = 0}^{m} {\cos (\alpha - \frac{2hk\pi }{{h_{s} n}})\cos (\frac{2k\pi }{n})}$$

7 $$GDSS{1}[i_{h} (t)] = G\sum\limits_{k = 0}^{m} {[\cos (a - 2kA) + \cos (a - 2kB)]}$$

where ${\text{G = I}}_{{\text{h}}} {/}({\text{m + }}1)$;$\alpha { = }h\omega t + \varphi_{h}$;$A = (h - h_{s} )*\pi /h_{s} n$;$B = (h + h_{s} )*\pi /h_{s} n$. According to the certain sums of trigonometric functions provided in reference [29], Eq. (7) can be further simplified as follows:

8 $$GDSS1[i_{h} (t)] = \left\{ {\begin{array}{*{20}c} {\begin{array}{*{20}c} {G(CD\csc A + EF\csc B),} & {h \ne h_{i} } \\ \end{array} } \\ {\begin{array}{*{20}c} {I_{h} \cos \alpha + GEF\csc B,} & {h = h_{s} (jn + 1)} \\ \end{array} } \\ {\begin{array}{*{20}c} {I_{h} \cos \alpha + GCD\csc A,} & {h = h_{s} (jn - 1)} \\ \end{array} } \\ \end{array} } \right.$$

where $C = \cos (\alpha - mA)$;$D = \sin [(m + 1)A]$;$E = \cos (\alpha - mB)$;$F = \sin [(m + 1)B]$;$h_{i} = h_{s} (jn \pm 1)$; *j* represents an arbitrary natural number; csc is a trigonometric function defined as the reciprocal of the sine function, expressed as $\csc \left( x \right) \, = \, 1/\sin \left( x \right)$. The same derivation process can be applied to analyze Eq. (5) and a similar expression can be obtained as follows:

9 $$GDSS2[i_{h} (t)] = \left\{ {\begin{array}{*{20}c} {\begin{array}{*{20}c} {G(JD\csc A + KF\csc B),} & {h \ne h_{i} } \\ \end{array} } \\ {\begin{array}{*{20}c} {I_{h} \sin \alpha + GKF\csc B,} & {h = h_{s} (jn + 1)} \\ \end{array} } \\ {\begin{array}{*{20}c} {I_{h} \sin \alpha + GJD\csc A \cdot } & {h = h_{s} (jn - 1)} \\ \end{array} } \\ \end{array} } \right.$$

where $J = \sin (\alpha - mA)$;$K = \sin (\alpha - mB)$. Observing Eqs. (8) and (9), it can be concluded that when *D* and *F* are both zero, *GDSS1* operator will exhibit a zero gain for any harmonic components at *h* ≠ *h*_i_, but a unity gain and zero phase shift for harmonic components at *h* = *h*_i_, including the selected harmonic order *h*_*s*_. The same conclusion can be drawn for the *GDSS2* operator except that it will introduce an extra -*π*/2 phase shift to the harmonics at *h* = *h*_i_.

When extracting the harmonic order to be detected, it is necessary to select the values of parameters *h*_*s*_, *m* and *n*. The value of *h*_*s*_ is related to the order of harmonic signal to be detected. Therefore, for an arbitrary integer value of *h*, *D* and* F* can both be zero simultaneously only if the values of *m* and *n* satisfy the integer condition specified in Eq. (10):

10 $$\frac{{(m + 1)*(h \pm h_{s} )}}{{h_{s} n}}$$

Considering that *m* is an integer smaller than *n*, the solution where both *D* and *F* are zero is given by:

11 $$m = h_{s} n - 1$$

The value of *n* determines the harmonic orders that can’t be filtered out by the two operators. Therefore, the selection of *n* must be based on the dominant harmonic components present in the input signal, as well as the result from Eq. (8) where *h*_*i*_ = *h*_*s*_(*jn* ± 1), in order to effectively avoid the harmonics that cannot be filtered out.

Table 2 presents the selection of parameters *m* and *n* for extracting different detected harmonic orders *h*_*s*_, along with the corresponding calculated values of *h*_*i*_. It is evident that with a reasonable selection of parameters *h*_*s*_, *m* and *n*, the *GDSS1* and *GDSS2* operators can effectively extract the desired harmonic components from the input signal, and the maximum delay time for *GDSS1* and *GDSS2* is *mT*/(*h*_*s*_*n*). For example, if the fundamental component is to be extracted, set *h*_*s*_ = 1. Using Eq. (10) and Eq. (11), along with the constraints on *h*_*i*_, the values of *m* = 14 and *n* = 15 are obtained.

**Table 2:** Relationships among *m*, *n*, *h*_*s*_ and *h*_*i*_*.*

*h*_*s*_	*m*	*n*	*h*_*i*_
1	14	15	1, 14, 16, 29, 31…
3	14	5	3, 12, 18, 27, 33…
5	14	3	5, 10, 20, 25, 35…
7	20	3	7, 14, 28…
4	15	4	4, 12, 20, 28, 36…
8	23	3	8, 16, 32…

**Fig. 3:**
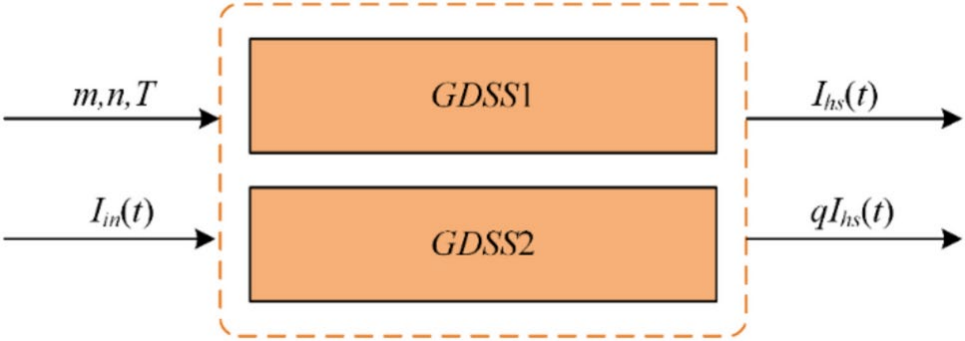
Illustrates of GDSS-based QSG method.

Both *GDSS1* and *GDSS2* operators have strong frequency selection characteristics, and the extracted two signals are orthogonal to each other. Therefore, they can be configured as a quadrature signal generation (QSG) algorithm, which is called the quadrature signal generation algorithm based on general delay signal superposition operators (GDSS-QSG) in this paper. Figure 3 illustrates the QSG algorithm based on the GDSS operators, where *I*_*h*s_ represents the *h*_*s*_-order harmonic component to be detected from the input signal *I*_in_ and *q* = *e*^-*jπ/*^^2^ denotes a *π/*2 phase delay operator.

### Frequency domain characteristic analysis

To further observe the filtering capabilities of the GDSS-QSG, Eq. (4) and Eq. (5) are converted to frequency domain by Laplace transform. The transfer functions of *GDSS1* and *GDSS2* are:

12 $$H_{GDSS1} (s) = \frac{2}{m + 1}\sum\limits_{k = 0}^{m} {e^{{ - \frac{kT}{{h_{s} n}}s}} } \cos \left( {\frac{2k\pi }{n}} \right)$$

13 $$H_{GDSS2} (s) = \frac{2}{m + 1}\sum\limits_{k = 0}^{m} {e^{{ - \frac{kT}{{h_{s} n}}s}} } \sin \left( {\frac{2k\pi }{n}} \right)$$

**Fig. 4:**
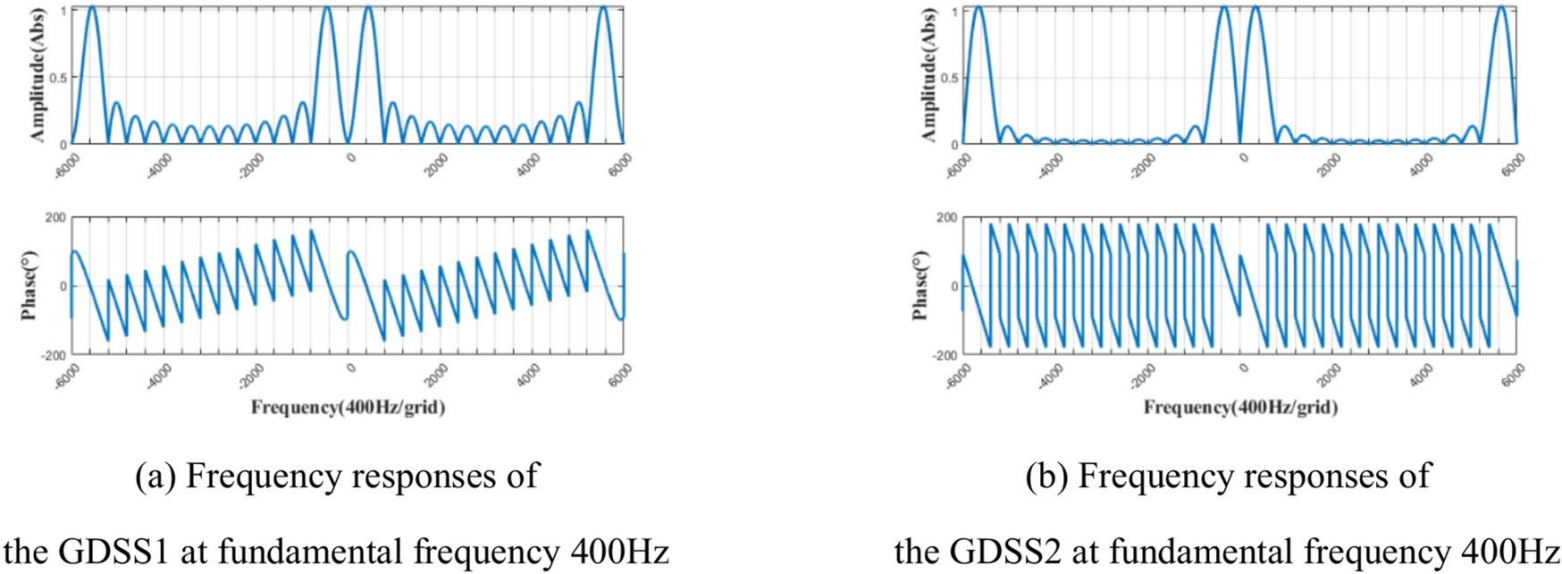
Frequency responses of the GDSS-QSG at fundamental frequency 400 Hz.

Assuming the grid fundamental frequency *f*_0_ = 400 Hz and *T* = 1/*f*_0_, the GDSS-QSG parameters are chosen as *h*_*s*_ = 1, *m* = 14, and *n* = 15. The magnitude frequency and phase frequency characteristic curves of Eq. (12) and Eq. (13) are shown in Fig. 4.

As shown in Fig. 4(a), the frequency response of *GDSS1* operator exhibits a unit gain and zero phase shift at frequencies *f* = (15* k* ± 1)*f*_0_(*k* = 0,1,2,3……), and zero gain at frequencies *f* ≠ (15* k* ± 1)*f*_0_(*k* = 0,1,2,3……).This demonstrates that when *m* = 14 and *n* = 15, the *GDSS1* operator can accurately extract the harmonic sequence (15* k* ± 1)*f*_0_ from the characteristic sequence of the input signal. As shown in Fig. 4(b), the frequency response of *GDSS2* operator exhibits a unit gain and a phase shift of -*π*/2 at frequencies *f* = (15* k* ± 1)*f*_0_(*k* = 0,1,2, 3……), while exhibits zero gain at frequencies *f* ≠ (15* k* ± 1)*f*_0_(*k* = 0,1,2,3……). This demonstrates that when *m* = 14 and *n* = 15, the *GDSS2* operator can accurately extract the harmonic sequence (15* k* ± 1)*f*_0_ from the characteristic sequence of the input signal, while exhibiting a phase lag of *π*/2 relative to the *GDSS1* operator. The peak value of the amplitude frequency characteristic curves of GDSS1 and GDSS2 is about 1.029 (Abs), slightly deviating from the fundamental frequency. However, this deviation does not affect the operators’ ability to accurately extract the fundamental component.

It is worth noting that when *h*_*s*_ = 1, *m* = 14, and *n* = 15, the GDSS-QSG method shown in Fig. 3 can’t eliminate the influence of the 14th and 16th even-order harmonics. However, in the MEA variable frequency AC power generation system, the power electronic converters and nonlinear onboard loads primarily generate odd-order harmonics below the 25th harmonic, with very few even-order harmonics. Therefore, it can be considered that the GDSS-QSG algorithm is capable of extracting a pure fundamental component orthogonal signal while effectively suppressing the influence of harmonics.

From the above analysis, it is evident that the proposed GDSS operators exhibit mathematical completeness in extracting the fundamental component. By constructing a linear combination of the delayed signal and the input signal, the required harmonic components can be directly extracted with extremely high accuracy. When used for fundamental component extraction, the delay time of the GDSS operators is 14* T*/15, resulting in a relatively short dynamic response time. This effectively addresses the issue faced by some traditional harmonic current detection methods, which struggle to balance detection speed and accuracy when processing high-frequency, rapidly changing signals.

## Positive-sequence fundamental component extraction and harmonic detection based on GDSS

### Positive-sequence fundamental component extraction structure based on GDSS

In practical engineering applications, the GDSS-QSG operators for calculating the fundamental component is implemented using digital signal processing chips. Therefore, it is necessary to discretize Eq. (4) and Eq. (5). If the number of sampling points of the input signal *i*(*t*) within one fundamental period is *N*_*s*_, the discretized GDSS-QSG operators for extracting the fundamental component can be expressed as follows:

14 $$I_{1} [{\text{t}}i] = \frac{2}{15}\sum\limits_{k = 0}^{14} i [{\text{t}}i - \frac{k}{15}N_{s} ]{\text{cos}}(\frac{2k\pi }{{15}})$$

15 $$qI_{1} [{\text{t}}i] = \frac{2}{15}\sum\limits_{k = 0}^{14} i [{\text{t}}i - \frac{k}{15}N_{s} ]{\text{sin}}(\frac{2k\pi }{{15}})$$

Assuming the sampling frequency of the airborne high frequency active power filter is *f*_*s*_ = 100 kHz and the fundamental frequency is *f*_*0*_ = 400 Hz, the number of sampling points *N*_*s*_ is calculated as *N*_*s*_ = *f*_*s*_*/ f*_*0*_ = 250. In this case, the delay modules of the GDSS-QSG operators for fundamental extraction $N_{k} = kN_{s} /15$ (*k* = 0,1,……14) may result in fractional values. In this case, if the system performs calculations by truncating the fractional part and using only the integer *N*_*k*_, rounding errors will occur, leading to the GDSS-QSG operators unable to accurately extract the fundamental component from the input signal. Therefore, a finite impulse response (FIR) filter based on the Lagrange interpolation method is used to approximate fractional order delays. Transformed the delay modules $z^{{{ - }N_{k} }}$ to ${\text{z}}^{{{\text{ - N}}_{{\text{k}}} }} {\text{ = z}}^{{{\text{ - N}}_{{\text{D + d}}} }}$, where *D* is the integer part and *d* is the fractional part. The FIR filter is used to approximate the fractional part of the delay modules.

According to Lagrange interpolation principle:

16 $$z^{ - d} \approx \mathop \sum \limits_{j = 0}^{n} \left[ {h(j)z^{ - j} } \right]$$

where *h*(*j*) is a polynomial coefficient, *j* = 0,1, ……*n*, The coefficient *h*(*j*) can be obtained from Eq. (17):

17 $$h(j) = \prod\limits_{{\begin{array}{*{20}c} {i = 0} \\ {i \ne k} \\ \end{array} }}^{n} {\frac{{{\text{d}} - i}}{j - i}}$$

where, *j* is the order of FIR filter, with a range of values from 0 to n. Table 3 shows the values of polynomial coefficients of FIR filters of order 1 to 3 based on Lagrange interpolation.

**Table 3:** FIR filter polynomial coefficient table.

	*h*(0)	*h*(1)	*h*(2)	*h*(3)
*n* = 1	1-*d*	*d*	/	/
*n* = 2	$\frac{{({\text{d}} - 1)({\text{d}} - 2)}}{2}$	$- {\text{d}}({\text{d}} - 2)$	$\frac{{{\text{d}}({\text{d}} - 1)}}{2}$	/
*n* = *3*	$- \frac{{({\text{d}} - 1)({\text{d}} - 2)({\text{d}} - 3)}}{6}$	$\frac{{{\text{d}}({\text{d}} - 2)({\text{d}} - 3)}}{2}$	$- \frac{{{\text{d}}({\text{d}} - 1)({\text{d}} - 3)}}{2}$	$\frac{{{\text{d}}({\text{d}} - 1)({\text{d}} - 2)}}{6}$

**Fig. 5:**
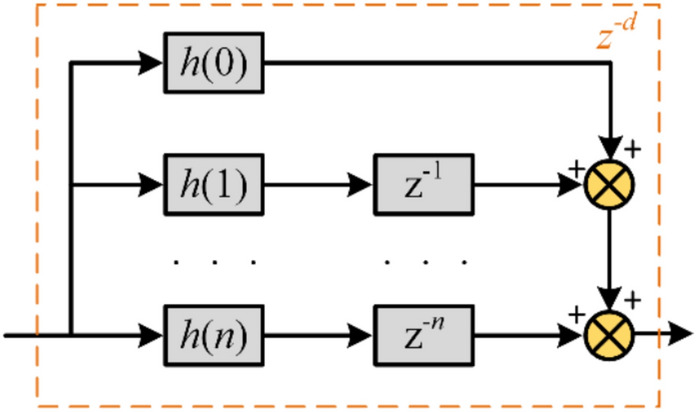
Structure diagram of FIR filter based on Lagrange interpolation method.

**Fig. 6:**
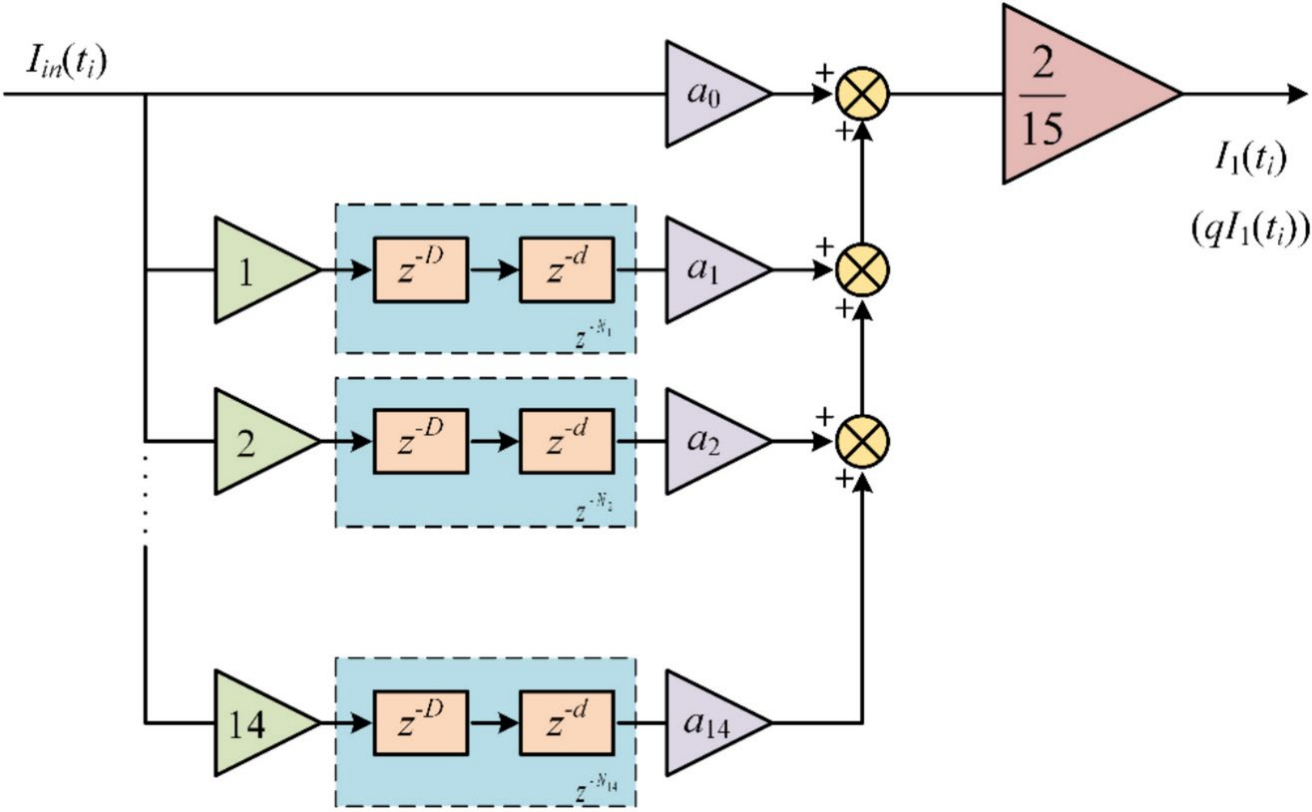
Implementation of GDSS-QSG structure.

Figure 5 shows the structure diagram of the FIR filter based on the Lagrange interpolation, while Fig. 6 illustrates the implementation form of the GDSS-QSG method proposed in this paper.

In Fig. 6, the parameters *a*_k_ in *GDSS1* and *GDSS2* operators are *cos*(2*kπ*/15) and *sin*(2*kπ*/15), respectively, *k* = 0,1,……14. By embedding the FIR filter into the delay modules, the GDSS-QSG operators are able to accurately extract the fundamental component even when *N*_*k*_ contains fractions, thus enhancing the accuracy of the operators.

**Fig. 7:**
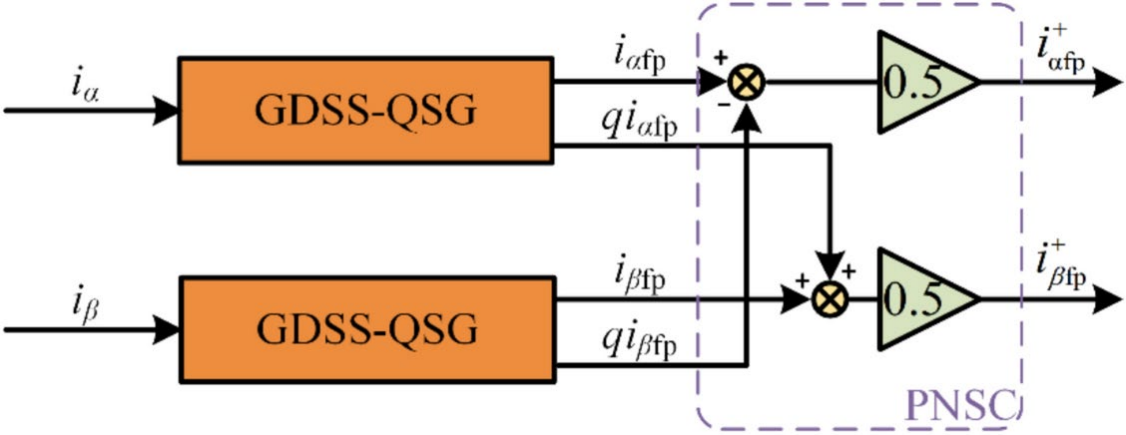
Block diagram of positive-sequence fundamental component extraction based on GDSS.

Figure 7 presents a block diagram for extracting the positive-sequence fundamental component using the GDSS method. As shown in the frequency response curves in Fig. 4, the GDSS-QSG operators simultaneously extract both the positive-sequence and negative-sequence components of the fundamental wave. However, the method does not allow for the selective extraction of the positive-sequence component alone. Therefore, it is necessary to perform Positive–Negative Sequence Separation (PNSC) on the extracted results. The derivation of the PNSC process is outlined in reference^28^, and the resulting expression can be directly applied in this study. According to reference^28^, the positive-sequence component in the *αβ* reference frame can be calculated as:

18 $$\begin{gathered} \left[ {\begin{array}{*{20}c} {I_{\alpha }^{ + } } \\ {I_{\beta }^{ + } } \\ \end{array} } \right] = \left[ {\begin{array}{*{20}c} {T_{\alpha \beta + } } \\ \end{array} } \right]\left[ {\begin{array}{*{20}c} {I_{\alpha } } \\ {I_{\beta } } \\ \end{array} } \right]; \hfill \\ \left[ {\begin{array}{*{20}c} {T_{\alpha \beta + } } \\ \end{array} } \right] = \frac{1}{2}\left[ {\begin{array}{*{20}c} {\begin{array}{*{20}c} 1 \\ q \\ \end{array} } & {\begin{array}{*{20}c} { - q} \\ 1 \\ \end{array} } \\ \end{array} } \right] \hfill \\ \end{gathered}$$

### Harmonic current detection algorithm based on GDSS

This paper primarily investigates the harmonic current detection algorithm applied to aviation APF onboard equipment, which operates within the variable-frequency-grids of MEA, with a frequency range of 380 to 800 Hz. Consequently, in addition to extracting the positive-sequence fundamental component, it is essential to incorporate a frequency detection step to ensure that the proposed algorithm is frequency-adaptive.

From the expressions of the GDSS-QSG operators during fundamental component extraction (as shown in Eq. (14) and Eq. (15)), it is evident that only *N*_*s*_ in the algorithm parameters is affected by changes in system frequency. When the system frequency varies, the delay modules in $z^{{{ - }N_{k} }}$ the GDSS-QSG must be adjusted accordingly to accurately extract the fundamental current from signals that vary in frequency.

To address this issue, a PLL is employed for real-time frequency tracking. Based on the frequency detection results from the PLL, *N*_*s*_ is updated in real-time, and the adaptive delay modules of the GDSS-QSG is dynamically adjusted. This approach ensures that the proposed harmonic current detection algorithm can track system frequency changes in real time and accurately extract the fundamental component, even under varying grid conditions.

Ideally, the grid voltage is a balanced three-phase sine wave. However, the operating conditions of the MEA’s variable-frequency-grids are often challenging, and various practical factors can cause grid voltage imbalances. To mitigate the impact of three-phase voltage unbalance on frequency detection, this study employs a decoupled double synchronous reference frame phase-locked loop (DDSRF-PLL)^29^. The DDSRF-PLL operates within a synchronous rotating reference frame, tracking the frequency by monitoring the positive-sequence components of the voltage. This method demonstrates excellent frequency adaptability, enabling accurate frequency detection even in the presence of grid voltage unbalance and frequency fluctuations.

**Fig. 8:**
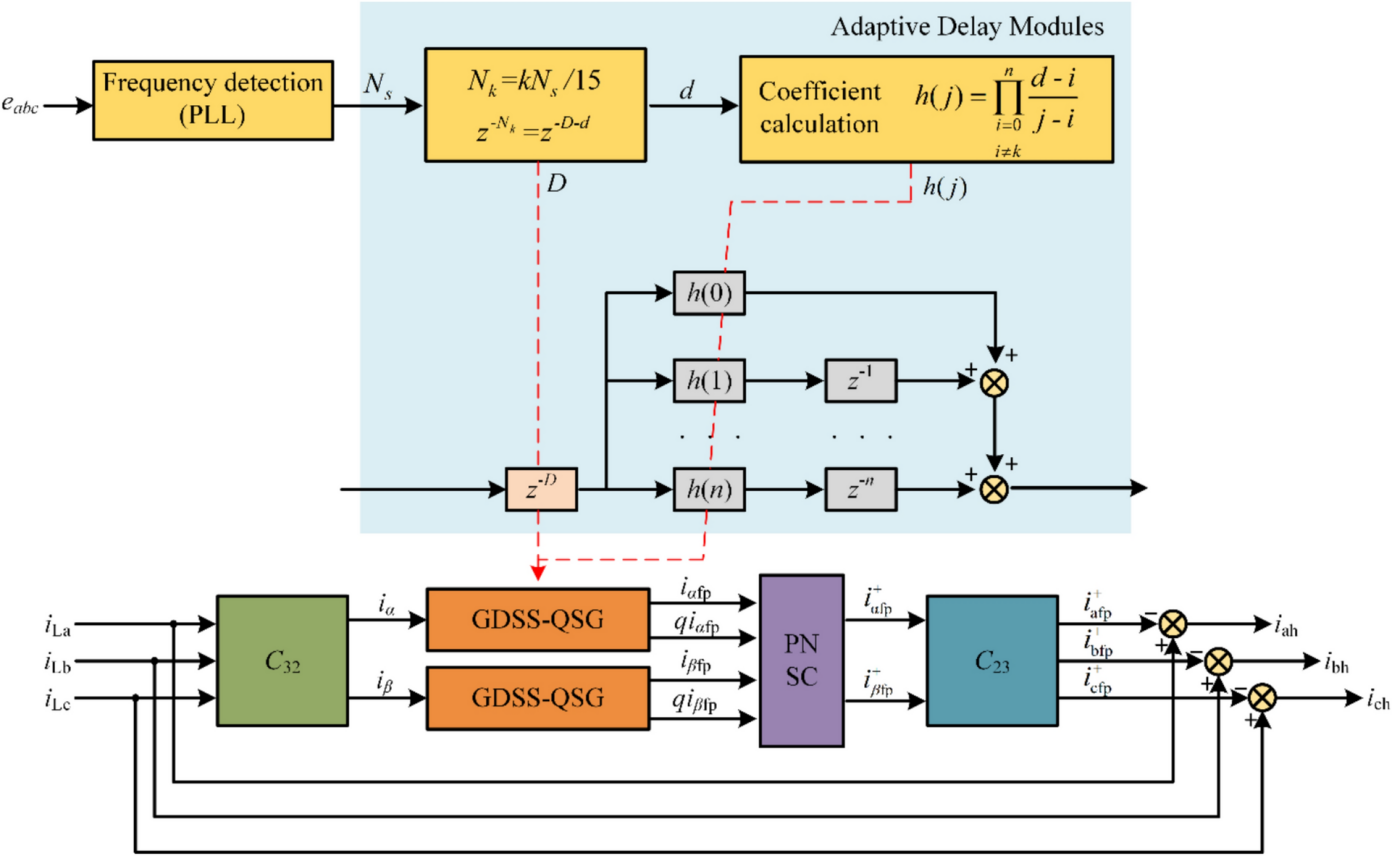
Block diagram of harmonic current detection algorithm based on GDSS.

The harmonic current detection algorithm based on GDSS proposed in this paper is illustrated in the block diagram shown in Fig. 8. In this structure, the GDSS-QSG operators incorporate adaptive delay modules, with the delay time varying according to the current system frequency.

**Fig. 9:**
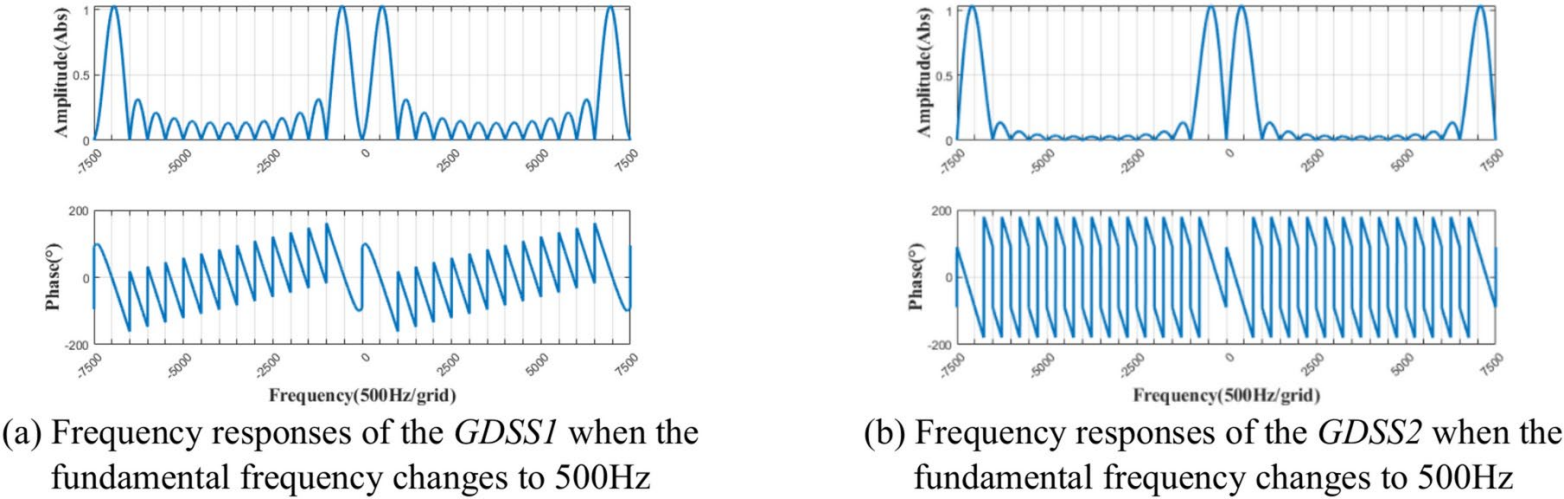
Frequency responses of the GDSS-QSG when the fundamental frequency changes to 500 Hz.

Assuming that the fundamental frequency of the power grid changes to *f*_*0*_^*’*^ = 500 Hz while the sampling frequency remains constant, the number of sampling points per period in the GDSS parameter will change accordingly. The new number of sampling points per period, denoted as *N*_*s*_^*’*^, can be calculated by $N_{s}^{\prime } = f_{s} / \, f_{0}^{\prime } = 200$. Figure 9 shows the frequency response of the *GDSS1* and *GDSS2* operators after frequency changes. It can be observed that after the frequency change, the harmonic sequence extracted by GDSS-QSG operators from the characteristic sequence of the input signal remains (15* k* ± 1)*f*_*0*_^*’*^. This evidences that positive-sequence fundamental component extraction structure based on GDSS using the adaptive delay modules can adapt to the change of the system frequency and complete the accurate extraction of the positive-sequence fundamental component.

By combining the GDSS operators with a PLL, the delay time can be dynamically adjusted without the need for complex parameter tuning, thereby maintaining high accuracy as the grid frequency fluctuates. This combination enhances the method’s robustness against frequency variations, making it well-suited for application in MEA’s variable-frequency-grids. The process of harmonic current detection algorithm based on GDSS is outlined as follows:

When the load current is distorted and the three phases are unbalanced, the load current *i*_*L*_ can be expressed as:

19 $$\left[ {\begin{array}{*{20}c} {i_{La} } \\ {i_{Lb} } \\ {i_{Lc} } \\ \end{array} } \right] = \left[ {\begin{array}{*{20}c} {\sum\limits_{n = 1}^{\infty } {\sqrt 2 } I_{an + } \sin \left( {n\omega t + \varphi_{n} } \right)} \\ {\sum\limits_{n = 1}^{\infty } {\sqrt 2 } I_{bn + } \sin \left( {n\left( {\omega t - \frac{2\pi }{3}} \right) + \varphi_{n} } \right)} \\ {\sum\limits_{n = 1}^{\infty } {\sqrt 2 } I_{cn + } \sin \left( {n\left( {\omega t + \frac{2\pi }{3}} \right) + \varphi_{n} } \right)} \\ \end{array} } \right] + \left[ {\begin{array}{*{20}c} {\sum\limits_{n = 1}^{\infty } {\sqrt 2 } I_{an - } \sin \left( {n\omega t + \varphi_{n} } \right)} \\ {\sum\limits_{n = 1}^{\infty } {\sqrt 2 } I_{bn - } \sin \left( {n\left( {\omega t + \frac{2\pi }{3}} \right) + \varphi_{n} } \right)} \\ {\sum\limits_{n = 1}^{\infty } {\sqrt 2 } I_{cn - } \sin \left( {n\left( {\omega t - \frac{2\pi }{3}} \right) + \varphi_{n} } \right)} \\ \end{array} } \right]$$

20 $$\left\{ {\begin{array}{*{20}l} {i_{{{\text{L}}.{\text{a}}}} = i_{{{\text{L}}a + }} + i_{{{\text{L}}a - }} } \hfill \\ {i_{{{\text{L}}b}} = i_{{{\text{L}}b + }} + i_{{{\text{L}}b - }} } \hfill \\ {i_{{{\text{L}}c}} = i_{{{\text{L}}c + }} + i_{{{\text{L}}c - }} } \hfill \\ \end{array} } \right.$$

where, *i*_*La*_、*i*_*Lb*_、*i*_*Lc*_ are the load currents of each phase respectively; *I*_*an*+_、*I*_*bn*+_、*I*_*cn*+_ are the effective values of positive-sequence components of each load current respectively; *I*_*an-*_、*I*_*bn-*_、*I*_*cn-*_ are the effective values of negative-sequence components of each load current respectively; *n* is the harmonic number; *φ*_*n*_ is the initial phase. First, the load current *i*_*L*_ is transformed from the three-phase stationary reference frame to the two-phase *αβ* reference frame, resulting in *i*_*Lα*_、*i*_*Lβ*_, using the Clarke transformation.

21 $$\left[ {\begin{array}{*{20}c} {i_{{{\text{L}}\alpha }} } \\ {i_{{{\text{L}}\beta }} } \\ \end{array} } \right] = {\varvec{C}}_{32} \left[ {\begin{array}{*{20}c} {i_{{{\text{La}}}} } \\ {i_{{{\text{Lb}}}} } \\ {i_{{{\text{Lc}}}} } \\ \end{array} } \right] = \left[ {\begin{array}{*{20}c} {\sum\limits_{n = 1}^{\infty } {\sqrt 3 } I_{\alpha n + } \sin \left( {n\omega t + \varphi_{n} } \right)} \\ {\sum\limits_{n = 1}^{\infty } {\sqrt 3 } I_{\beta n + } \sin \left( {n\omega t + \varphi_{n} } \right)} \\ \end{array} } \right] + \left[ {\begin{array}{*{20}c} {\sum\limits_{n = 1}^{\infty } {\sqrt 3 } I_{{\alpha n{ - }}} \sin \left( {n\omega t + \varphi_{n} } \right)} \\ {\sum\limits_{n = 1}^{\infty } {\sqrt 3 } I_{{\beta n{ - }}} \sin \left( {n\omega t + \varphi_{n} } \right)} \\ \end{array} } \right]$$

where $${\varvec{C}}_{32} = \sqrt{\frac{2}{3}} \left[ {\begin{array}{*{20}c} 1 & { - \frac{1}{2}} & { - \frac{1}{2}} \\ 0 & {\frac{\sqrt 3 }{2}} & { - \frac{\sqrt 3 }{2}} \\ \end{array} } \right]$$; *I*_*αn*+_、*I*_*βn*+_ are positive-sequence components of load current in two-phase *αβ* reference frame respectively; *I*_*αn-*_、*I*_*βn-*_ are negative-sequence components of load current in two-phase *αβ* reference frame. When the load currents *i*_*Lα*_ and *i*_*Lβ*_ in the *α* and *β* axes are processed through the GDSS-QSG for filtering and positive negative-sequence separation, the corresponding positive-sequence fundamental components *i*^+^_*αfp*_ and *i*^+^_*βfp*_ in the two-phase reference frame can be obtained. Subsequently, these components can be transformed back to the three-phase stationary reference frame using the inverse Clarke transformation, resulting in the three-phase positive-sequence fundamental currents *i*^+^_*afp*_, *i*^+^_*bfp*_, and *i*^+^_*cfp*_.

22 $$\left[ {\begin{array}{*{20}c} {i_{afp}^{ + } } \\ {i_{bfp}^{ + } } \\ {i_{cfp}^{ + } } \\ \end{array} } \right] = C_{23} \left[ {\begin{array}{*{20}c} {i_{\alpha fp}^{ + } } \\ {i_{\beta fp}^{ + } } \\ \end{array} } \right] = \left[ {\begin{array}{*{20}c} {\sqrt 2 I_{a1 + } \sin \left( {\omega t + \varphi_{1} } \right)} \\ {\sqrt 2 I_{b1 + } \sin \left( {\omega t - \frac{2\pi }{3} + \varphi_{1} } \right)} \\ {\sqrt 2 I_{c1 + } \sin \left( {\omega t + \frac{2\pi }{3} + \varphi_{1} } \right)} \\ \end{array} } \right]$$

where ***C***_23_ is the inverse matrix of ***C***_32_; *I*_*a*1+_、*I*_*b*1+_、*I*_*c*1+_ are the effective values of positive-sequence current of each fundamental component respectively. Finally, the three-phase harmonic currents *i*_*ah*_、*i*_*bh*_、*i*_*ch*_ to be detected can be obtained by subtracting the three-phase positive-sequence fundamental currents *i*^+^_*afp*_、*i*^+^_*bfp*_、*i*^+^_*cfp*_ from the three-phase load currents *i*_*L*_.

## Experimental validation

To verify the superior performance of the harmonic current detection algorithm based on GDSS in adverse power grid conditions, this paper comprehensively considered the influence of current harmonics, three-phase unbalance and frequency jump on the performance of harmonic detection algorithm, compared and analyzed the filtering performance, dynamic performance and robustness of the proposed algorithm with traditional harmonic detection method based on *i*_*p*_*-i*_*q*_ method^12^, MAF method^17^, ANF method^19^ and SAI method^20^.

In the practical application of multi-electric aircraft, hardware resources are typically limited. Therefore, it is essential to evaluate the computational complexity of the proposed algorithm before conducting experiments. By observing the discrete expressions of the GDSS-QSG operators in Eq. (14) and Eq. (15), it can be inferred that GDSS-QSG operators consists of two parts: the number of sampling points within a single fundamental period and the mathematical operations after fixing parameters. Among them, the trigonometric function part can be pre calculated and stored. Therefore, the process of the GDSS- QSG operators can simply be realized with 2(*m* + 1) multiplications and 2* m* additions. According to Fig. 8, the process of proposed algorithm mainly requires to compute two GDSS-QSG operators and a PNSC. Consequently, the proposed detection method requires a total of 62 multiplications, 58 additions. Actually, considering the sliding window update strategy and hardware parallel acceleration, the mathematical operations of the GDSS operators can be further reduced. In summary, the processing time of the proposed algorithm is beneficial for the real-time operation of the control system.

In the comparison methods, the *i*_*p*_*-i*_*q*_ method typically uses second-order LPFs with a fixed cutoff frequency, resulting in the lowest computational complexity. MAF takes the average of the number of sampling points within a single sampling period, while SAI multiplies and integrates the input signal with the reference signal, resulting in a lower computational complexity than the proposed algorithm. ANF uses adaptive algorithms for filtering, which requires dynamic adjustment of weights and has a higher computational complexity than the proposed algorithm. Although the proposed algorithm has a high computational complexity, it can meet the requirements of real-time feasibility and still has advantages in MEA’s variable-frequency grid power system which require both detection accuracy and dynamic response speed.

According to the experimental modeling of MEA loads and FFT analysis of load currents in references [12,30] and [31], the MEA’s variable-frequency-grids mainly contains fifth and seventh harmonics. In the experiment, three sets of sine signal generation modules containing fundamental and harmonic were used to simulate the phase A, phase B, and phase C of the load current. To reproduce the real conditions of variable-frequency-grids of the MEA, experiments were conducted using a simulated harmonic source under four distinct operating conditions. The experimental conditions were designed as follows:

Condition 1 Simulates a load current with a fixed frequency of 400 Hz under balanced three-phase conditions. The amplitudes of phases A, B, and C are all 10 A with positive-sequence phases, and harmonic content consists of 10% for the fifth and seventh harmonics.

Condition 2 simulates load current with a fixed frequency of 600 Hz and unbalanced three phase conditions. The amplitude of phase A is 5A, phase B is 10A, and phase C is 15A, and content consists of 10% for the fifth and seventh harmonics.

Condition 3 simulates load current under small frequency variations and balanced three phases. The amplitudes of phases A, B, and C are all 10A with a positive-sequence phase, and content consists of 10% for the fifth and seventh harmonics. The system frequency transitions from 400 to 380 Hz.

Condition 4 simulates load current under large frequency variations and three-phase unbalance. The amplitude of phase A is 5A, phase B is 10A, and phase C is 15A, and content consists of 20% for the third harmonic, 15% for the fifth harmonic, and 10% for the seventh harmonic. The system frequency transitions from 800 to 750 Hz. Table 4 shows the load current parameters under different working conditions, in the condition 2 and condition 4, the amplitude of the positive sequence fundamental component contained in the three-phase unbalanced load current is 10A. Table 5 presents the model parameters for different harmonic current detection algorithms.

**Table 4:** Load current parameters under different under different working conditions.

Condition	Phase A		Phase B		Phase C		Operating frequency (Hz)	Frequency after change (Hz)	Harmonic content (%)		
	Amplitude(A)	Phase(rad)	Amplitude(A)	Phase(rad)	Amplitude(A)	Phase(rad)			Third harmonic	Fifth harmonic	Seventh harmonic
1	10	0	10	-2*π*/3	10	2*π*/3	400	400	0	10	10
2	5	0	10	-2*π*/3	15	2*π*/3	600	600	0	10	10
3	10	0	10	-2*π*/3	10	2*π*/3	400	380	0	10	10
4	5	0	10	-2*π*/3	15	2*π*/3	800	750	20	15	10

**Table 5:** Parameters for different detection methods.

Detection algorithms	Parameters
GDSS-QSG	*h*_*s*_ = 1, *m* = 14, *n* = 15, linear Lagrange interpolation
LPF	Damping coefficient *ζ* = 0.707, cutoff frequency of 400 Hz
MAF	Sliding window length *T*_*w*_ = 2*π/ω*
ANF	Damping coefficient *ζ* = 0.707, adaptation gain *γ* = 20
SAI	Center angular frequency 2*πf*_*0*_

**Fig. 10:**
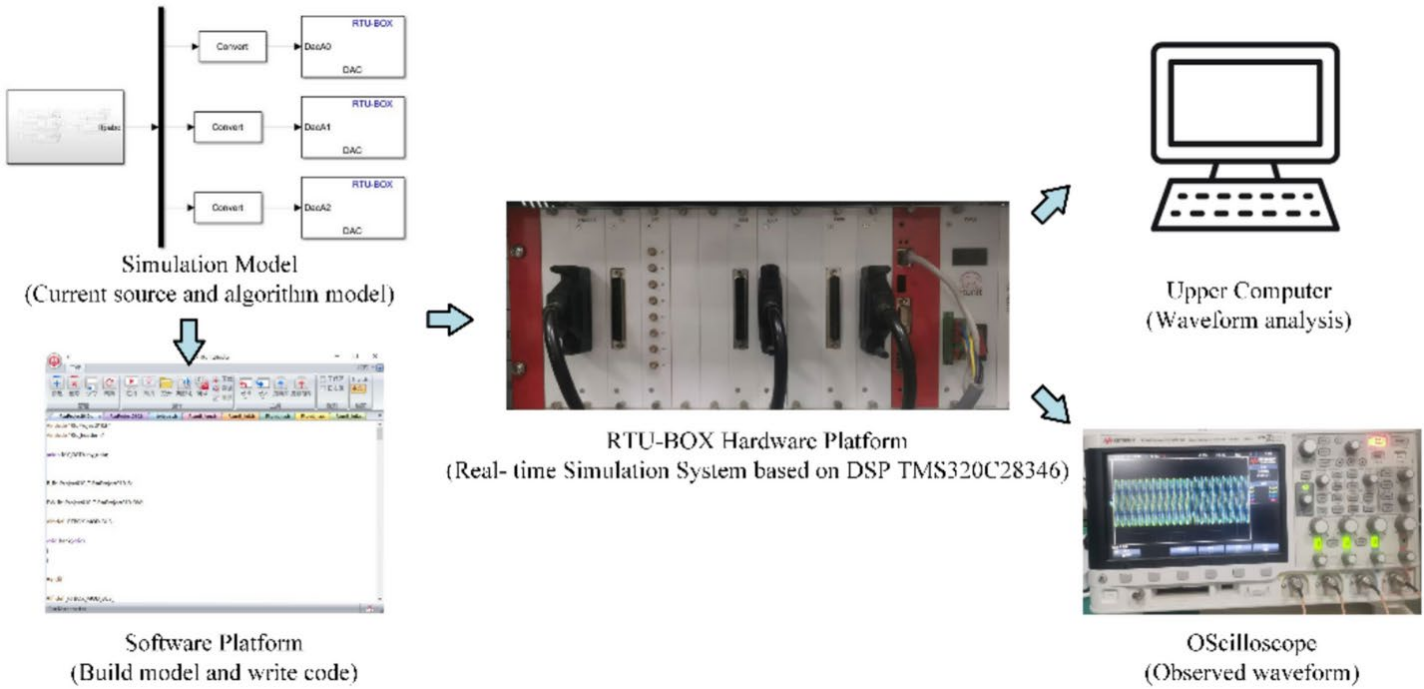
Experimental process.

Experiments were conducted on the RTU-BOX Real-Time Simulation System based on DSP TMS320C28346. Simulink based algorithm models can be directly generated into code and downloaded to hardware, and then form a loop with hardware to complete real-time operation and rapid verification of control code. The experimental process is illustrated in Fig. 10. First, the RTU-Toolbox library in Simulink is utilized to construct a simulation model of the current sources and the harmonic current detection algorithms. Next, the simulation models are converted into a C language program using the RTU-BOX software platform, and the program is subsequently downloaded to the RTU-BOX hardware platform. Finally, the program’s operation is monitored in real time using an oscilloscope, and the waveform data is transferred to the upper computer for FFT analysis. The sampling frequency used during the experiment was 15 kHz.

### Condition 1 experimental analysis

**Fig. 11:**
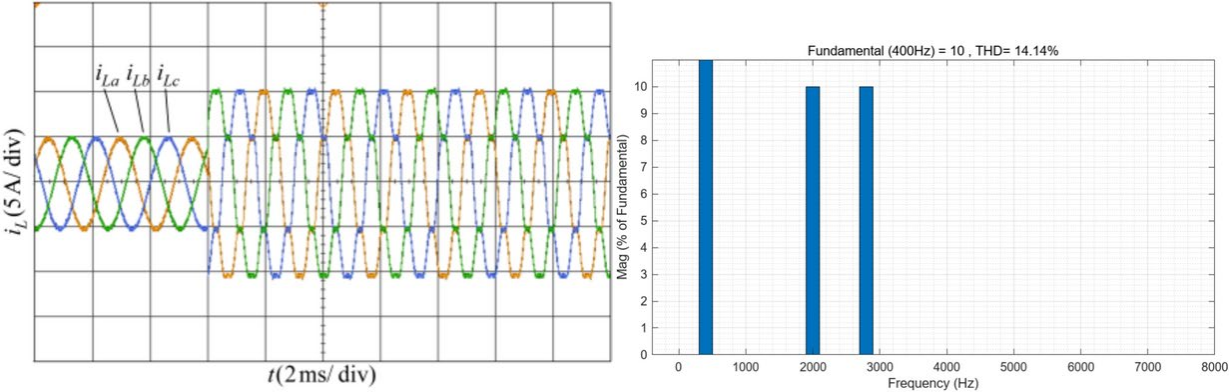
Current waveform and FFT analysis under condition 1.

The system operates at a frequency of 400 Hz with balanced three phase simulated currents. Figure 11 shows the simulated current waveform and the FFT analysis results of phase A current. It can be observed that after 6 ms, the THD of the phase A load current is 14.14%.

**Fig. 12:**
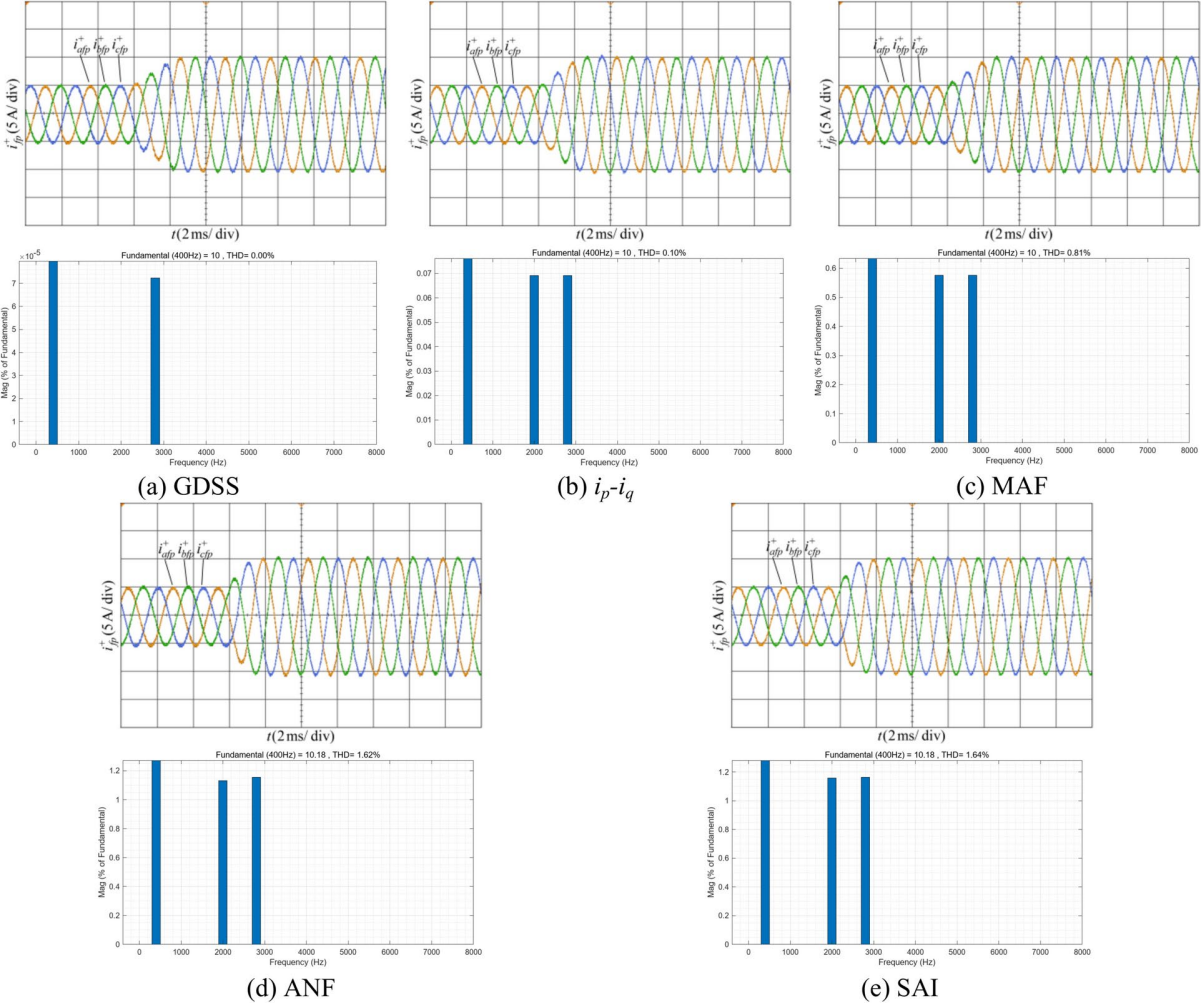
Positive-sequence fundamental current waveform extracted by various detection methods and FFT analysis in condition 1.

Figure 12 shows the proposed harmonic current detection algorithm and the comparison detection methods for extracting positive-sequence fundamental current and FFT analysis results under condition 1. It can be observed that when the current contains only a small amount of harmonic content, all detection methods demonstrate satisfactory filtering performance. Among these methods, the proposed GDSS algorithm, *i*_*p*_*-i*_*q*_ method, and detection method based on MAF achieve 100% accuracy in the amplitude of the positive-sequence fundamental current, while detection method based on ANF and SAI are slightly less accurate, with an accuracy of 98.2%. In terms of THD, the proposed GDSS algorithm outperforms the other methods, with a THD value close to zero, indicating superior filtering performance. In terms of dynamic response time, the proposed detection method achieves a response time of 14/15 fundamental periods, the shortest among all the compared methods. In contrast, the *i*_*p*_*-i*_*q*_ method, which utilizes a low-pass filter, exhibits the longest response time of approximately 1.5 fundamental periods. The other detection methods, including those based on MAF, ANF, and SAI, demonstrate a response time of one fundamental period.

### Condition 2 experimental analysis

**Fig. 13:**
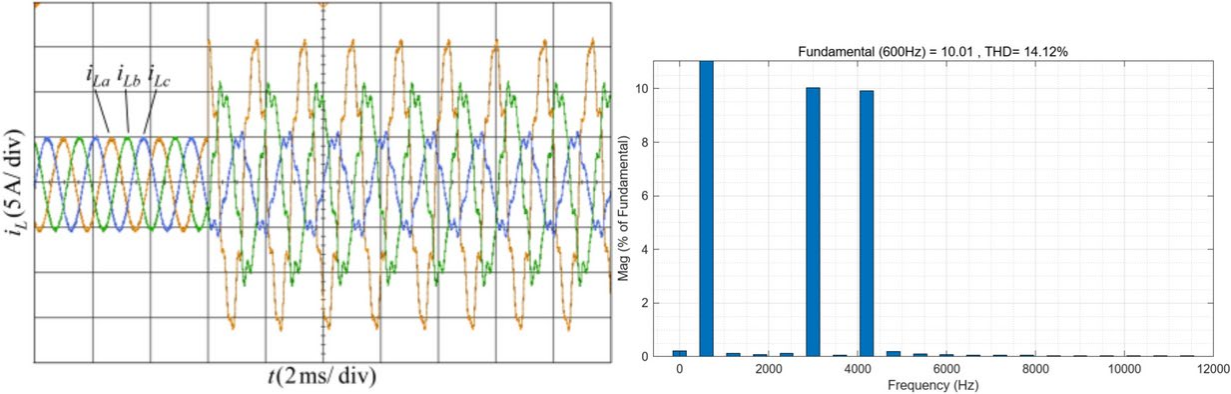
Current waveform and FFT analysis under condition 2.

The system frequency is 600 Hz. At 6 ms, a three-phase unbalance occurs in the simulated current. The amplitude of phase A is 5A, phase B is 10A, and phase C is 15A. At the same time, 10% of the fifth and seventh harmonics are added. The simulated current waveform and the FFT analysis results of the positive-sequence current A phase current after phase sequence calculation are shown in Fig. 13. It can be observed that the THD of phase A load current is 14.12%.

**Fig. 14:**
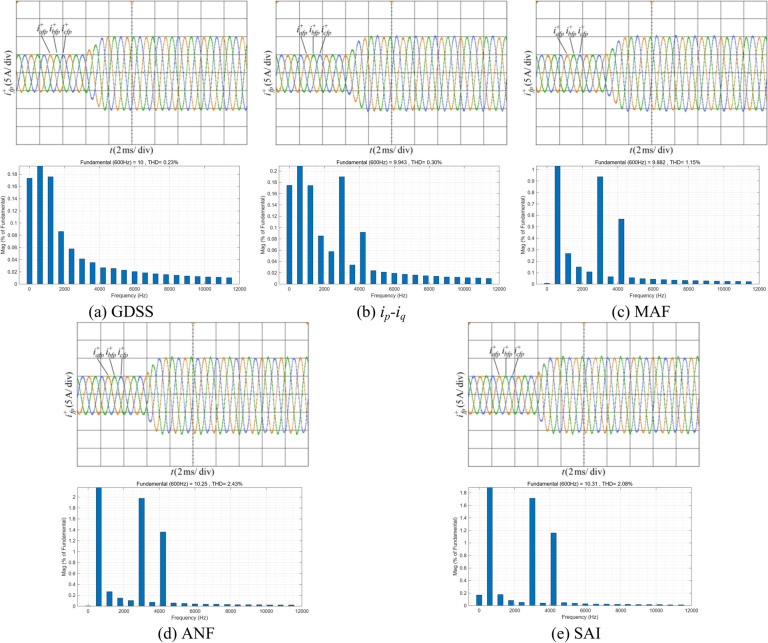
Positive-sequence fundamental current waveform extracted by various detection methods and FFT analysis in condition 2 .

Figure 14 shows the proposed harmonic current detection algorithm and the comparison detection methods for extracting fundamental positive-sequence current and FFT analysis results under condition 2. It can be seen that after adding three-phase unbalance, the proposed GDSS detection algorithm returned to normal after a brief adjustment. After stabilization, the three-phase unbalance had almost no impact on the detection of the positive-sequence fundamental component. In contrast, the other detection methods are significantly affected by the three-phase unbalance, resulting in a slight decrease in detection accuracy.

### Condition 3 experimental analysis

**Fig. 15:**
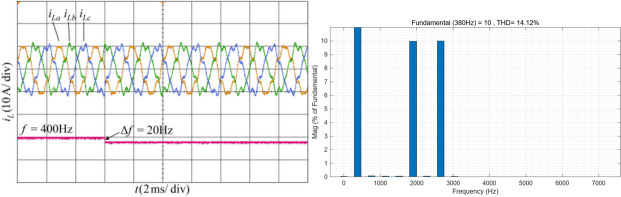
Current waveform and FFT analysis under condition 3.

The system operates at a frequency of 400 Hz with balanced three phase simulated currents, containing 10% of the fifth and seventh harmonics. At 6 ms, a frequency jump occurs and the frequency changes to 380 Hz. The simulated current waveform and the FFT analysis results of phase A current are shown in Fig. 15. The THD of the phase A load current is 14.12%.

**Fig. 16:**
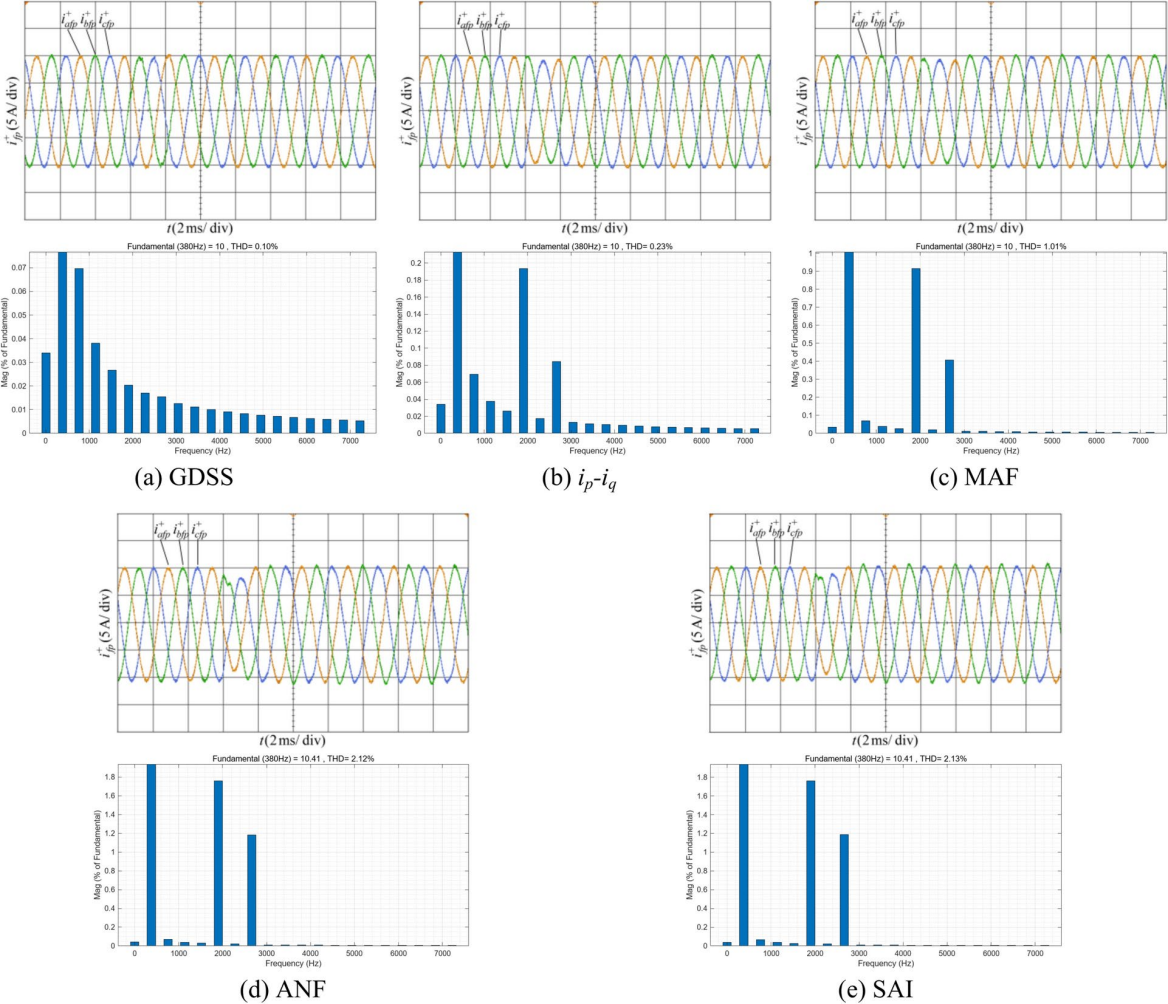
Positive-sequence fundamental current waveform extracted by various detection methods and FFT analysis in condition 3.

Figure 16 shows the proposed harmonic current detection algorithm and the comparison detection methods for extracting fundamental positive-sequence current and FFT analysis results under condition 3.

It is evident that the proposed GDSS detection algorithm completes the positive-sequence fundamental components detection after the frequency jump the fastest, with a short adjustment time, smooth transition process, and the highest detection accuracy. In contrast, due to the inherent cutoff frequency of the low-pass filter, the performance of the* i*_*p*_*-i*_*q*_ method detection method decreases and the dynamic time is long. The MAF detection method can dynamically adjust the window length based on frequency changes, but the detection time is still one fundamental period. For detection method based on ANF and SAI, although these methods can adapt to frequency variations, their filtering capabilities are limited. Compared to other detection methods, the proposed GDSS detection algorithm demonstrates superior performance under conditions of frequency variation.

### Condition 4 experimental analysis

**Fig. 17:**
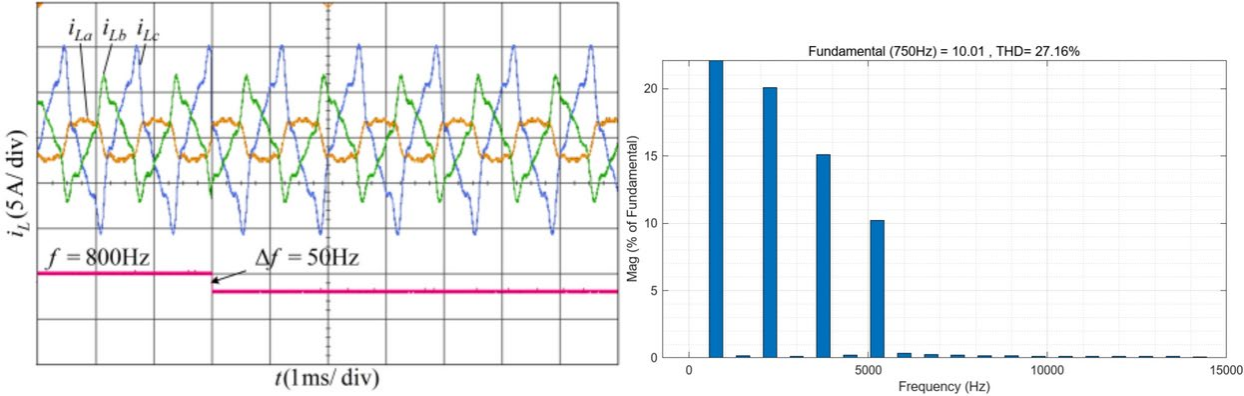
Current waveform and FFT analysis under condition 4.

The system operates at a frequency of 800 Hz with unbalanced three phase simulated currents, containing of 20% third, 15% fifth, and 10% seventh harmonics. At 3 ms, a frequency jump occurs, changing the frequency to 750 Hz. The simulated current waveform and the FFT analysis results of the positive-sequence current A phase current after phase sequence calculation are shown in Fig. 17. It can be observed that the THD of phase A load current is 27.16%. Among all operating conditions, the power environment under this condition is the most complex and can rigorously test the performance of harmonic current detection methods.

**Fig. 18:**
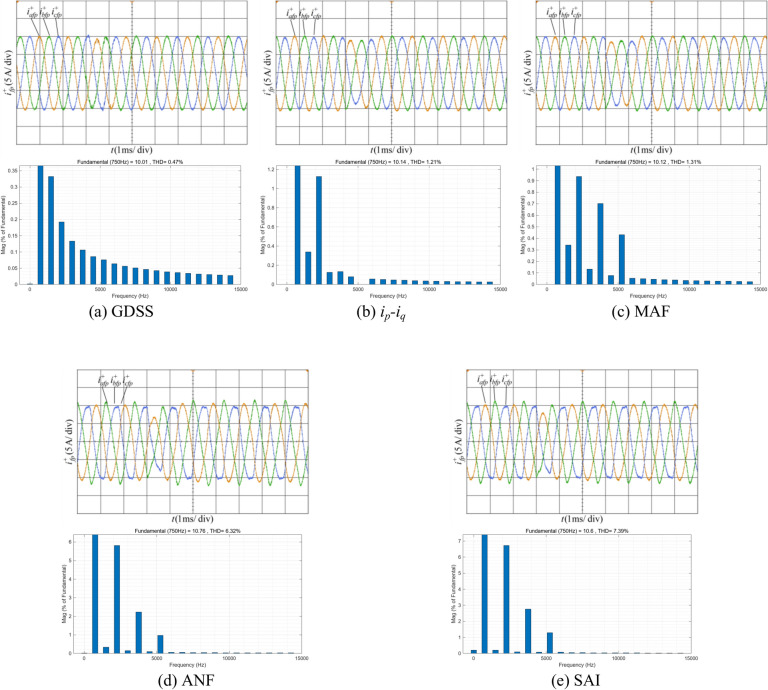
Positive-sequence fundamental current waveform extracted by various detection methods and FFT analysis in condition 4.

Figure 18 shows the proposed harmonic current detection algorithm and the comparison detection methods for extracting fundamental positive-sequence current and FFT analysis results under condition 4.

The results demonstrate that the proposed GDSS detection algorithm exhibits strong robustness, maintaining superior filtering performance and dynamic response under complex conditions. It accurately detects the positive-sequence fundamental current within one fundamental period following a frequency jump, achieving a positive-sequence amplitude error of 0.01% and a THD of 0.47%, both of which are the lowest among the detection methods. In contrast, the *i*_*p*_*-i*_*q*_ method and detection method based on MAF have good robustness, they are less effective than the proposed algorithm in terms of detection accuracy and response speed. The ANF and SAI detection methods yield high THD for the positive-sequence fundamental component due to degraded filtering performance in complex power grid environments, leading to poor robustness.

Table 6 presents the results of detection accuracy, filtering performance of all harmonic current detection algorithms under different experimental conditions. These results are presented in the form of accuracy percentages for the positive-sequence fundamental current extraction, as well as the THD of the detected signal. Table 7 presents the results of dynamic response time and robustness of all harmonic current detection algorithms under different experimental conditions. The results demonstrate that, compared to other methods, the proposed GDSS detection algorithm exhibits superior detection accuracy, enhanced filtering performance, and a faster dynamic response time. Especially under challenging conditions such as severe harmonic interference, three-phase imbalance, and frequency jump typical of aviation power grids, The proposed method demonstrates robust performance, ensuring continuous and reliable detection under various operating conditions.

**Table 6:** Detection accuracy and filtering performance of different detection algorithms under different experimental conditions.

Experimental condition	Detection algorithm	Amplitude	THD	Amplitude accuracy
Condition 1	Proposed GDSS	10.00A	0.00%	100%
	*i*_*p*_*-i*_*q*_	10.00A	0.10%	100%
	MAF	10.00A	0.81%	100%
	ANF	10.18A	1.62%	98.2%
	SAI	10.18A	1.64%	98.2%
Condition 2	Proposed GDSS	10.00A	0.23%	100%
	*i*_*p*_*-i*_*q*_	9.94A	0.30%	99.4%
	MAF	9.88A	1.15%	98.85%
	ANF	10.25A	2.43%	97.57%
	SAI	10.31A	2.08%	97.92%
Condition 3	Proposed GDSS	10.00A	0.10%	100%
	*i*_*p*_*-i*_*q*_	10.00A	0.23%	100%
	MAF	10.00A	1.01%	100%
	ANF	10.41A	2.12%	95.9%
	SAI	10.41A	2.13%	95.9%
Condition 4	Proposed GDSS	10.01A	0.47%	99.9%
	*i*_*p*_*-i*_*q*_	10.14A	1.21%	98.6%
	MAF	10.12A	1.31%	98.8%
	ANF	10.76A	6.32%	92.4%
	SAI	10.60A	7.39%	94.0%

**Table 7:** Dynamic response time and robustness of different detection algorithms.

Detection algorithm	Dynamic response time	Robustness
Proposed GDSS	14* T*/15	Strong
*i*_*p*_*-i*_*q*_	1.5* T*	Good
MAF	*T*	Good
ANF	*T*	Poor
SAI	*T*	Poor

## Conclusion

This paper begins by deriving the GDSS operators and analyzing its discrete form in practical applications. The accuracy of the operator’s calculation is improved through Lagrange interpolation. Subsequently, a PLL and a positive–negative sequence separation module are integrated with the GDSS operator to achieve accurate harmonic current extraction in environments with variable frequency and three-phase unbalanced grids. Finally, experiments were designed to validate the proposed algorithm under various operating conditions. The main conclusions drawn from the experimental results are as follows:

The proposed harmonic current detection algorithm based on the GDSS is capable of accurately extracting harmonic currents within a single fundamental cycle, with a dynamic response time of 14 T/15. The proposed algorithm simultaneously offers high detection accuracy and fast response speed, making it well-suited for MEA’s variable-frequency-grids, which demand both rapid detection and precise harmonic current extraction.

The proposed harmonic current detection algorithm based on GDSS demonstrates exceptional robustness, enabling it to quickly and accurately extract harmonic currents even in complex aviation environments. Compared to other methods, the proposed harmonic current detection algorithm based on GDSS achieves rapid and accurate synchronization under complex conditions, highlighting its exceptional capability in variable-frequency-grids of MEA.

While the proposed detection algorithm exhibits impressive performance, it does have limitations regarding computational complexity. Future work can focus on optimizing the operators to reduce the computational burden on the hardware.

## Data Availability

The datasets used and/or analysed during the current study available from the corresponding author on reasonable request.

## References

[1] Barzkar, A. & Ghassemi, M. Components of electrical power systems in more and all-electric aircraft: a review[J]. *IEEE Trans. Transport. Electric.* **8**(4), 4037–4053 (2022).

[2] Barzkar, A. & Ghassemi, M. Electric power systems in more and all electric aircraft: a review. *IEEE Access.* **8**, 169314–169332 (2020).

[3] Schefer, H. et al. Discussion on electric power supply systems for all electric aircraft. *IEEE Access.* **8**, 84188–84216 (2020).

[4] S. Liang, *et al*. Overview and analysis of electric power systems for more/all electric aircraft. In *IECON 2023- 49th Annual Conference of the IEEE Industrial Electronics Society* 1–6 (2023).

[5] Dai, Z., Yang, J., Rao, D., Zhang, J. & Zhang, Z. A global convergence estimator of grid voltage parameters for more electric aircraft. *IEEE Trans. Industr. Electron.* **67**(9), 7540–7549 (2020).

[6] Patnaik, B., Kumar, S. & Gawre, S. Recent advances in converters and storage technologies for more electric aircrafts: a review. *IEEE J. Miniaturization Air Space Syst.* **3**(3), 78–87 (2022).

[7] Zhu C, *et al*. An improved half-bridge type active power filter for aircraft power grids. In 2021 *7th International Conference on Systems and Informatics (ICSAI)* 1–6 (2021).

[8] Xu, Z. et al. A quadrature signal-based control strategy for Vienna rectifier under unbalanced aircraft grids. *IEEE J. Emerging Selected Topics Power Electron.* **10**(5), 5280–5289 (2022).

[9] Eid, A., El-Kishky, H., Abdel-Salam, M. & El-Mohandes, M. T. On power quality of variable-speed constant-frequency aircraft electric power systems. *IEEE Trans. Power Delivery* **25**(1), 55–65 (2010).

[10] A. Mukherjee & C. S.. Cascaded SOGI-FLL based reference current extraction method for active power filter in more electric aircraft. In *2020 IEEE 17th India Council International Conference (INDICON)* 1–5 (2020).

[11] Y. Gao, *et al*. Research on APF based on average current value instantaneous reactive power harmonic detection. In *2024 IEEE 7th Information Technology, Networking, Electronic and Automation Control Conference (ITNEC)* 1349–1352 (2024).

[12] Hao, Z., Wang, X. & Cao, X. Harmonic control for variable-frequency aviation power system based on three-level NPC converter. *IEEE Access.* **8**, 132775–132785 (2020).

[13] Kanjiya, P., Khadkikar, V. & El Moursi, M. S. Adaptive low-pass filter based DC offset removal technique for three-phase PLLs. *IEEE Trans. Industr. Electron.* **65**(11), 9025–9029 (2018).

[14] Babu, B. C. A novel adaptive bandpass filter based PLL for grid synchronization under distorted grid conditions. *IEEE Trans. Instrument. Measure.* **71**, 1–11 (2022).

[15] Freijedo, F. D., Doval-Gandoy, J., Lopez, Ó., Fernandez-Comesana, P. & Martinez-Penalver, C. A signal-processing adaptive algorithm for selective current harmonic cancellation in active power filters. *IEEE Trans. Industr. Electron.* **56**(8), 2829–2840 (2009).

[16] Robles, E. et al. Frequency-adaptive stationary-reference-frame grid voltage sequence detector for distributed generation systems. *IEEE Trans. Industr. Electron.* **58**(9), 4275–4287 (2011).

[17] G. Panda, S. Jena & P. Rangababu. A low voltage ride through scheme for three phase grid connected PV inverter with an adaptive window based MAF-PLL. In *2018 8th IEEE India International Conference on Power Electronics (IICPE)* 1–6 (2018).

[18] Yazdani, D., Bakhshai, A., Joos, G. & Mojiri, M. A real-time three-phase selective-harmonic-extraction approach for grid-connected converters. *IEEE Trans. Industr. Electron.* **56**(10), 4097–4106 (2009).

[19] Yazdani, D., Bakhshai, A. & Jain, P. K. A three-phase adaptive notch filter-based approach to harmonic/reactive current extraction and harmonic decomposition. *IEEE Trans. Power Electron.* **25**(4), 914–923 (2010).

[20] Y. Zhaoyang, *et al*. Full current harmonic detection method based on sinusoidal amplitude integrator. In *IECON 2017 - 43rd Annual Conference of the IEEE Industrial Electronics Society* 1172–1179 (2017).

[21] Chen, M., Peng, L., Wang, B. & Wu, W. Accurate and fast harmonic detection based on the generalized trigonometric function delayed signal cancellation. *IEEE Access.* **7**, 3438–3447 (2019).

[22] Lavopa, E., Zanchetta, P., Sumner, M. & Cupertino, F. Real-time estimation of fundamental frequency and harmonics for active shunt power filters in aircraft electrical systems. *IEEE Trans. Industr. Electron.* **56**(8), 2875–2884 (2009).

[23] V. I. Suryawanshi & N. N. Jangle. Precise measurement of power system frequency and phasor using SDFT and comparison with DFT based approach. In 2017 *IEEE International Conference on Power, Control, Signals and Instrumentation Engineering* (ICPCSI) 402–407 (2017).

[24] Yang, B. et al. Improvement of recursive DFT for APF with higher switching frequency to suppress wideband harmonics. *IEEE Access.* **9**, 144300–144312 (2021).

[25] Selvajyothi, K. & Janakiraman, P. A. Extraction of harmonics using composite observers. *IEEE Trans. Power Delivery* **23**(1), 31–40 (2008).

[26] G. Li, X. Wen & Z. Wang. Harmonic detection of power grid based on the optimal reduced-order observer. In *2*023 *42nd Chinese Control Conference (CCC)* 7206–7211 (2023).

[27] Gradshteyn, I. S. & Ryzhik, I. M. *Equation of referencing in Table of Integrals, Series, and Products 36* (Academy Press, 2007).

[28] Rodríguez, P. et al. Multiresonant frequency-locked loop for grid synchronization of power converters under distorted grid conditions. *IEEE Trans. Industr. Electron.* **58**(1), 127–138 (2011).

[29] Z. Feng, et al. Research on the performance of DDSRF-PLL under unbalanced grid voltage. In 2023 *CAA Symposium on Fault Detection, Supervision and Safety for Technical Processes (SAFEPROCESS)* 1–5 (2023).

[30] Z. Tai, , *et al*. AC quantity calculation of wide variable frequency electric power system for aircraft with recursive DFT. In 2016 Chinese Control and Decision Conference (CCDC) 2776–2781 (2016).

[31] H. Nademi & Z. Soghomonian. Performance characteristics of a multilevel active power filter with optimal predictive control for more-electric-aircraft concept. In 2016 *International Conference on Electrical Systems for Aircraft, Railway, Ship Propulsion and Road Vehicles & International Transportation Electrification Conference* (ESARS-ITEC) 1–7 (2016).

